# Endovascular Neurosurgery in the Netherlands: Historical Developments and Achievements

**DOI:** 10.3389/fsurg.2018.00054

**Published:** 2018-09-20

**Authors:** Joost de Vries, Peter Willems, Hieronymus Boogaarts, André Grotenhuis

**Affiliations:** ^1^Neurosurgery, Radboud University Nijmegen Medical Centre, Nijmegen, Netherlands; ^2^Neurosurgery, Leiden University Medical Center, Leiden, Netherlands

**Keywords:** endovascular neurosurgery, history, Netherlands, aneurysm, flow diverter

## Abstract

The historical developments of endovascular neurosurgery in the Netherlands are described.

Endovascular Neurosurgery in The Netherlands started in 1992 when André Grotenhuis at Radboudumc, Nijmegen studied the effect of balloon-expandable stents in the treatment of experimental carotid aneurysms (1). In this study, first a glass model of aneurysms was constructed and Palmaz-Schatz ^TM^ stents were inserted. Flow characteristics were evaluated with dye. In the second part of the study 10 experimental aneurysms of the cervical carotid artery in beagles were treated with stents. Interestingly, the positioning of the stents was evaluated with angioscopy, as the stents were barely visible under fluoroscopy. All 10 aneurysms occluded and all but one carotid artery remained patent.

In the early nineties at several academic centers in The Netherlands radiologists started to perform neuroendovascular procedures.

Political barriers obstructed the participation of neurosurgeons in this field.

In 2004, however, an agreement between the Departments of Neurosurgery and Radiology at Radboudumc Nijmegen was reached and Joost de Vries was able to start a neuroendovascular fellowship in Nijmegen. He completed this fellowship at Karolinska University Hospital, Stockholm, Sweden in 2006.

The first endovascular neurosurgical procedure in The Netherlands was performed in July 2006.

At that time, Cees Tulleken, well-known for his Excimer assisted high flow bypass surgery technique led his team at the frontier of neurovascular surgery, at the University Medical Center Utrecht (2). One of his residents, Peter Willems also developed an interest for endovascular neurosurgery.

In July 2008, immediately after finishing his neurosurgical residency, Peter Willems left for Toronto, Canada, to do a clinical interventional neuroradiology fellowship with Karel TerBrugge. Upon his return to the Netherlands, 1 year later, he took a combined neurosurgical and radiological position at the Leiden University Medical Center.

More or less simultaneously, Jeroen Boogaarts, neurosurgical resident in Nijmegen, followed an endovascular neurosurgery fellowship with Joost de Vries. After finishing this fellowship in 2010 he continued to work in Nijmegen in both open vascular and endovascular neurosurgery.

Although endovascular neurosurgery was pioneered in the early nineties by Bernd Richling and Andreas Gruber in Vienna and Alexander Andreou in Athens, performing endovascular procedures by neurosurgeons in Europe remained somewhat controversial. Therefore, a web-based prospective database was constructed in 2007 at Radboudumc Nijmegen in order to obtain objective data on neurovascular treatment results. Careful evaluation of outcomes disclosed that treatment of aneurysm by dual trained neurosurgeons was safe (3). Jeroen Boogaarts went on to publish his thesis on “Quality of care for aneurysmal subarachnoid hemorrhage. From theoretical considerations to practical implementations” (4).

Further research efforts developed when, in 2010, Ajay Wakhloo was looking for a site in Europe to perform a clinical study for his Surpass flow diverter, after having elaborated the concept of flow diversion in aneurysms. This resulted in a pivotal safety and feasibility study, conducted in Nijmegen, including 10 patients. As a result of this initial experience, a training center for flow diversion was initiated at Radboudumc Nijmegen and more than 120 training sessions for over 250 neurointerventionalists from all over the world have been given since. Peter Willems started research on the use of time resolved CTA (4D-CTA) in neurovascular arteriovenous shunts during his year in Toronto and continued these efforts in Leiden after his return (5, 6). The elucidation of the diagnostic validity of 4D-CTA and its value in disorders like pulsatile tinnitus were further explored in collaboration with the Nijmegen group. This collaboration continues to thrive, moving its focus predominantly toward optimal clinical treatment strategies for intracranial aneurysms.

Besides this clinical research, the group in Nijmegen has begun to focus on the complex mechanism of aneurysm wall inflammation and break down, causing rupture. This is studied in various animal models (7).

Since The Netherlands is a relatively small country (around 17 million inhabitants in an area less than a third of that of the state of New York), nationwide research initiatives are also feasible and the MRCLEAN trial, looking into the endovascular treatment of acute ischemic stroke, is a very successful recent example of this (8). Endovascular neurosurgeons also participate in such initiatives, demonstrating the cooperation between the different medical specialties involved in these treatments.

Due to the growing awareness that neurovascular diseases were being treated by different specialties, whose efforts would benefit from nationwide collaboration, 2015 saw the birth of the Dutch Neurovascular Society (“Netherlands Neurovasculair Genootschap”). This society brought together neurovascular neurosurgeons, vascular neurologists, and neurointerventional radiologists. Within the context of this society, the abovementioned pioneering led to the gradual acceptance of non-radiologists in the field of neurointerventional procedures in The Netherlands. The Dutch Neurovascular Society developed and approved training standards for neuroendovascular training and, thus, further opened up the neurointerventional field to non-radiologists such as neurologists and neurosurgeons. As a result, 5 Dutch neurologists/neurosurgeons have now followed a neuroendovascular training program at 5 different Dutch neurointerventional sites. In addition, young neurosurgeons from abroad (Italy and Germany) have also done a hybrid neurovascular fellowship at the Radboudumc in Nijmegen.

In order to monitor, and eventually improve, quality of care for neurosurgical patients in The Netherlands, the Dutch Society of Neurosurgery initiated a nationwide registry, (Quality Registry Neurosurgery), in 2010. This was spearheaded by Jeroen Boogaarts and one of the four patient subgroups in this registry is the group of patients who suffered a subarachnoid hemorrhage. The results of endovascular neurosurgery are among the many outcome parameters being evaluated with this database. Such initiatives demonstrate the ability of medical specialists, from different specialties and from different medical centers, within a country to combine forces in an effort to improve the quality of their health care program.

Especially with the increasing need for (emergency) endovascular treatments, such collaboration also becomes increasingly important within one center or one region. In many centers in The Netherlands, neurointerventionalists have combined forces with body interventionalists to allow a 24/7 coverage for stroke treatment. Consequently, one can come across interventional neurologists, interventional neurosurgeons, interventional neuroradiologists, and/or interventional body-radiologists working side-by-side, in the angiosuite. In some parts of the world, this would not be surprising, but in Europe this is still quite a unique situation.

Despite these developments, it remains difficult to envision what lies ahead. Due to lengthy training programs and persisting political barriers for cross-training, it is still not self-evident for a neurosurgeon to enter the neuroendovascular field. And once a neurosurgeon has been trained successfully, he/she requires a position where both open and endovascular neurosurgery may be performed. This requires cooperation from two departments in the appointment of such an individual as in The Netherlands any X-ray-guided procedure falls under the responsibility of the Department of Radiology. In The Netherlands, there has not yet been a precedent where such an individual decided to dedicate him-/herself fully to endovascular treatment. Therefore, it is unclear what the implications of such a decision would be for his/her license to practice.

According to the authors more neurosurgeons should be dual-trained and the political barriers need to be removed for the following reasons.

A young Dutch neurosurgeon interested in the neurovascular field can be trained in open neurovascular techniques without any political barrier. As the case-load in most, if not all Dutch Neurosurgical Centers, however, has become so low (mean number of acute clippings per center per year is 18; no data available for clipping of unruptured aneurysms) it is questionable whether enough experience can be obtained in open vascular neurosurgery. Beside that the efficiency of an open neurosurgeon being on call just for clipping is very low considering the case-load. It seems that this discourages young neurosurgeons for choosing the neurovascular subspecialty. This can be solved either by further centralization of neurovascular neurosurgery or by dual-training or by doing both. It is the experience of the authors and of others that dual-training clearly leads to cross-pollination and thus improvement of the skills in both endovascular and open neurovascular techniques (9).

Dual-trained neurosurgeons can offer a more efficient and less expensive 24/7 emergency service for neurovascular diseases as compared to a service where this is performed by interventional radiologists and open only neurosurgeons.

As there are no results yet from the above-mentioned Dutch database we cannot shed light whether a dual-trained individual is better or an open-only neurosurgeon.

As of 2017 there are 3 dual trained interventional neurosurgeons in The Netherlands among a total of 26 practicing neurointerventionalists.

## Author contributions

All authors listed have made a substantial, direct and intellectual contribution to the work, and approved it for publication.

### Conflict of interest statement

The authors declare that the research was conducted in the absence of any commercial or financial relationships that could be construed as a potential conflict of interest.
